# The effects of physical exercise on college students’ subjective wellbeing: the chain mediating role of perceived social support and psychological resilience

**DOI:** 10.3389/fpsyg.2026.1853782

**Published:** 2026-05-20

**Authors:** Shihao Wang, Feng Tai, Guoxing Zhang, Hangqi Liu

**Affiliations:** School of Physical Education, Liaoning Normal University, Dalian, China

**Keywords:** chain mediation, perceived social support, physical exercise, psychological resilience, subjective wellbeing

## Abstract

**Introduction:**

This study aims to explore the mechanism of physical exercise’s influence on the subjective wellbeing of contemporary college students, and focuses on the mediating role of perceived social support and psychological resilience in it.

**Methods:**

Through a large-scale questionnaire survey of 513 college students, a chain mediation model was constructed, and the mediating effects of perceived social support and psychological resilience were examined and analyzed using structural equation modeling and Bootstrap method.

**Results:**

(1) Physical exercise can positively predict college students’ subjective wellbeing; (2) Physical exercise can mediate college students’ subjective wellbeing through perceived social support and psychological resilience, respectively; (3) Physical exercise can affect college students’ subjective wellbeing through perceived social support and psychological resilience in a chain. Three pathways of mediating effects: physical exercise → perceived social support → subjective wellbeing (path 1), physical exercise → psychological resilience → subjective wellbeing (path 2), and physical exercise → perceived social support → psychological resilience → subjective wellbeing (path 3).

**Discussion:**

The results of the study show that physical exercise not only directly promotes subjective wellbeing, but also indirectly affects subjective wellbeing through the mediating effect of perceived social support and psychological resilience. This study provides empirical evidence for an in-depth understanding of the relationship between physical exercise and subjective wellbeing, and provides theoretical references for the synergistic promotion of college students’ mental health and academic development.

## 1 Introduction

As society accelerates and the pressures of academic and social adaptation increase, university students face growing psychological challenges that significantly affect their mental health and wellbeing. As a key indicator of mental health, subjective wellbeing plays a crucial role in mitigating negative emotions during this critical developmental stage. The relationship between physical exercise and subjective wellbeing has garnered significant attention in physical education and positive psychology. Although numerous studies have explored this relationship, the underlying mechanisms through which physical exercise enhances subjective wellbeing remain underexplored (Zhou and Zhou, 2022; Xu, 2014). This gap highlights the need for further research to identify the key factors influencing subjective wellbeing and the processes through which these factors interact. Although many studies indicate that physical activity interventions can enhance subjective wellbeing (Oh et al., 2025; Ying and Yang, 2025), others suggest that physical exercise may have no effect or even a negative impact in certain populations (Netz et al., 2005; Qu and Yao, 2017). These findings underscore the complexity of the relationship between physical exercise and wellbeing, suggesting that the effects of exercise may be mediated by various psychological variables (Marques et al., 2025; Zhao et al., 2024; Chen and Yu, 2015). Research into mediating factors, such as loneliness, social support, self-efficacy, and motivation, has identified their partial mediating roles in the exercise-wellbeing relationship (Diaz and Abbas, 2025; Liu, 2016). However, these studies do not fully account for the broader psychological processes at play. Existing research has shown that psychological resilience helps individuals mobilize resources, facilitating greater social support and enhancing subjective wellbeing (Hunter and Chandler, 1999; Rueger et al., 2010). Perceived social support has been linked to reduced stress and enhanced mental wellbeing, playing a vital role in the development of psychological resources such as self-efficacy and self-esteem (Tao and Li, 2025; Wu et al., 2017). Despite the substantial body of research on physical exercise and subjective wellbeing among university students, the literature remains fragmented in several ways (Xu and Fang, 2021; Ang et al., 2026). Many studies focus primarily on direct effects or examine isolated mediators such as self-efficacy or motivation, without offering an integrated perspective on how multiple psychological resources interact. A more comprehensive understanding of the mechanisms is essential, as subjective wellbeing is widely recognized as the result of dynamic interactions between external social resources and internal psychological capacities (Zhao et al., 2023; Li et al., 2023). However, the coordination between these dimensions, particularly the sequential relationship between perceived social support and psychological resilience, has not been sufficiently explored. This lack of a unified framework for understanding these interactions has practical implications. Current interventions targeting student wellbeing often address physical activity, social support, and psychological adaptation in isolation, which may limit their effectiveness (Su and He, 2023; Qiu et al., 2023). To optimize interventions, it is crucial to identify how these factors interact within an integrated model. Therefore, the present study aims to fill this gap by constructing and testing a chain mediation model that links physical exercise with perceived social support and psychological resilience, ultimately enhancing subjective wellbeing. By integrating both external and internal pathways, this study seeks to offer a more comprehensive framework for understanding the mechanisms through which physical exercise influences wellbeing to provide targeted suggestions for promoting the overall development of college students’ physical and psychological health.

## 2 Chain mediation hypothesis model of the relationship between physical exercise and subjective wellbeing

### 2.1 The effect of physical exercise on college students’ subjective wellbeing

Subjective wellbeing refers to an individual’s overall assessment of their quality of life based on self-defined criteria, encompassing both emotional and cognitive aspects; it is an important comprehensive psychological indicator for measuring an individual’s quality of life (Diener, 1984). Physical exercise enhances subjective wellbeing by providing participants with pleasurable and enjoyable experiences, thereby improving self-esteem, life satisfaction, interpersonal relationships and health benefits; furthermore, the higher the level of physical activity, the greater the subjective wellbeing (Qiao and Fan, 2020; Biwen et al., 2023). Furthermore, research indicates that the intensity and duration of physical exercise also influence subjective wellbeing, with long-term physical exercise having a significant effect on enhancing students’ subjective wellbeing; as the duration of low- and moderate-intensity exercise increases, its impact on students’ subjective wellbeing deepens, whilst the impact of high-intensity exercise on students’ subjective wellbeing becomes even more pronounced as the duration increases (Jiang and Bian, 2025; Diener et al., 1999). Physical exercise is by no means merely a form of physical activity; it is, in fact, a form of mental healing and a catalyst for social interaction. Adolescents with higher levels of subjective wellbeing experience more positive emotions, which encourages them to form social connections and, in turn, enhances their capacity for prosocial behavior (Fredrickson, 1998). For university students, physical exercise integrates direct physiological pleasure, positive psychological development and rich social support, thereby nurturing subjective wellbeing in a holistic manner. The reason physical activity brings about a sense of wellbeing is that it inherently contains elements conducive to happiness, namely play, leisure, games, exercise, competition, and spectator education. A number of big data-based observational studies have used instrumental variable methods to verify a possible causal relationship between physical exercise and subjective wellbeing (Wicker and Frick, 2015). Subjective wellbeing comprises three dimensions: life satisfaction, positive emotions and negative emotions (Chen and Yu, 2015). Physical exercise enables individuals to achieve physical and mental satisfaction and pleasure, thereby enhancing their subjective evaluation of life satisfaction (Diener, 2009). It can improve positive emotions and reduce negative emotions, consistent with the findings of previous scholars (Tan et al., 2020; Zhang and Zhou, 2013). Therefore, the research hypothesis H1: Physical exercise positively predicts college students’ subjective wellbeing is proposed.

### 2.2 Mediating effects of perceived social support

Perceived social support refers to subjective social support (Barrera, 1986); it is an individual’s subjective assessment of the extent of support they perceive, as well as their level of satisfaction with the support received (Zimet et al., 1988). Research conducted among university students has found that physical exercise has a positive effect on the level of perceived social support (Dorak, 2015), and that the amount of physical exercise is significantly positively correlated with perceived social support. Physical exercise helps students make friends and expand their social networks, thereby strengthening their social support; the more they exercise, the broader their social network resources become, and the more support they receive. Research indicates that there is a significant correlation between perceived social support and individual wellbeing. Physical exercise enhances university students’ perceived social support, thereby increasing their subjective wellbeing (Feng et al., 2012), and has a positive predictive effect on wellbeing (Wang and Zhang, 2015). The higher an individual’s perceived level of social support, the higher their level of subjective wellbeing; however, the magnitude of the influence varies across different dimensions, with support from friends and family having the greatest impact on subjective wellbeing. The mechanisms underlying the role of perceived social support primarily comprise two aspects: the direct mechanism and the buffering mechanism. Research into the direct mechanism has shown that perceived social support among university students has a direct positive predictive effect on positive emotions, thereby promoting and enhancing individuals’ subjective wellbeing (Liao et al., 2023). Some research on buffering mechanisms suggests that the perception of social support has a positive buffering effect on negative emotions such as anxiety, depression and distress in individuals, preventing the worsening of these negative emotions and providing a sustained regulatory effect on mental health and perceived wellbeing. Individuals with a high capacity for perceiving social support are better able to recognize the support available from friends, family and other sources. When faced with danger or difficulties, they are more proactive in resolving problems, making more effective use of perceived support, and promoting the transformation and understanding of positive emotions, thereby enhancing their level of subjective wellbeing. One of the functions of social support is to alleviate an individual’s anxiety and depression through interaction with others (Song and Fan, 2013). Engaging in social interactions with a focus on perceiving social support can significantly reduce an individual’ s anxiety levels, thereby fostering a sense of wellbeing (Wang et al., 2020). Physical exercise, as a social activity and behavior, involves social interactions that give rise to certain perceptions and insights. The level of social support individuals perceive can predict their level of subjective wellbeing, and this mechanism also influences their perception of risk in a similar way. When university students engage in physical exercise, they perceive an increase in the level of social support, which in turn enhances their subjective wellbeing. Therefore, the research hypothesis H2: Physical exercise positively predicts college students’ subjective wellbeing through the mediating role of perceived social support is proposed.

### 2.3 Mediating effects of psychological resilience

Mental toughness (also known as resilience) is an individual’s ability or trait to cope with stress, setbacks and trauma (Davydov et al., 2010; Ruiz, 2012). As a positive personality trait and psychological capacity, psychological resilience not only helps individuals maintain a healthy mental state amidst stress and setbacks and cope better with life’s various uncertainties, but also enhances personal perseverance, determination and self-control, thereby promoting students’ personal growth and all-round development. These abilities all contribute to enhancing university students’ subjective wellbeing. In research on the development of psychological resilience, physical exercise is regarded as a significant influencing factor, with mechanisms including reducing physical and psychological responses to stress, improving an individual’s physiological and psychological state, and acting as a ‘buffer’ against stress (Belcher et al., 2021). Physical exercise does not directly reduce depressive symptoms, but it can alleviate them through the mediating effects of social support and psychological resilience (Yoshikawa et al., 2016). Some researchers have pointed out that individuals with high levels of psychological resilience not only cope effectively with setbacks, but also seek out opportunities and challenges for personal growth; they tend to focus on their goals, respond effectively to challenges, have confidence in their abilities, act decisively in interpersonal relationships, feel in control of their lives, and are able to regulate their emotional states (McGeown et al., 2017). It is evident, therefore, that psychological resilience represents an individual’s effective coping and positive adaptation to life, and is highly correlated with subjective wellbeing. Research in the field of sport indicates that there is a significant positive correlation between psychological resilience and physical exercise; as a protective resource, it influences wellbeing, and participation in high- and moderate-intensity physical exercise can effectively enhance university students’ mental health and levels of psychological resilience (Liu, 2020). The recreational, open-ended and competitive nature of physical activity provides an excellent environment for the development of psychological resilience among university students. Psychological resilience is closely linked to subjective wellbeing. Research has found that psychological resilience is positively correlated with life satisfaction and positive emotions, and negatively correlated with negative emotions; furthermore, the direct effect of psychological resilience on subjective wellbeing is significant (Eldeleklioglu and Yildiz, 2020). Furthermore, psychological resilience has been discussed as a mediating variable in numerous studies; for instance, it mediates the relationship between hope and mental health, as well as subjective wellbeing (Yıldırım and Arslan, 2022). Based on existing evidence, this study hypothesis that regular participation in physical exercise among university students will enhance their psychological resilience, enabling them to maintain psychological stability in stressful and frustrating situations. Consequently, they are expected to evaluate their life circumstances more positively and maintain positive emotional experiences. Therefore, the research hypothesis H3: Physical exercise positively predicts college students’ subjective wellbeing through the mediating role of mental toughness is proposed.

### 2.4 Chain mediation of perceived social support and psychological resilience

The above review indicates that physical exercise, perceived social support and psychological resilience each have a significant positive impact on students’ subjective wellbeing, and that perceived social support and psychological resilience each play a simple mediating role in the process by which physical exercise influences students’ subjective wellbeing. Research has found that there is a significant relationship between perceived social support and psychological resilience; a strong capacity for perceived social support can create favorable conditions for individuals to cope positively with various challenges (lijie et al., 2016). It has been recognized that social support can influence an individual’s subjective wellbeing directly, or indirectly through the mechanisms of self-esteem and psychological resilience. When students feel supported by their peers and the school, their perseverance and confidence are enhanced, and they overcome difficulties through practical action, thereby increasing their psychological resilience (Kaye-Kauderer et al., 2022), which in turn improves their subjective wellbeing and creates a positive cycle. Setbacks and failures in sport contribute to the development of psychological resilience in adolescents, whilst support from teammates, the ability to accept feedback, and realistic performance evaluations all serve as protective factors leading to positive adaptation (Murphy et al., 2022). Adolescents with a high level of perceived social support are better able to perceive and experience the practical assistance provided by others, society and the state; they are also better able to understand and interpret the significant role such practical assistance plays in their present or future development (Ye, 2005). They transform this practical assistance into internal resources to enhance their recognition of their own abilities and worth, thereby promoting the development of self-esteem and subjective wellbeing (Zhou et al., 2018). The higher a university student’s overall sense of wellbeing, the more likely they are to interpret others’ behavior as supportive. Certain rational behaviors, spiritual beliefs and positive interpretations of the meaning of life events exhibited by students with high levels of wellbeing may prompt them to actively seek coping resources, such as social support, thereby enabling them to cope effectively with stress. It has been recognized that social support not only acts as a buffer for individuals in stressful situations, reducing the emergence of negative emotions, but also directly enhances positive emotions, life satisfaction and subjective wellbeing (Jiaxi et al., 2022). Research has found that social support positively influences psychological resilience, and that social support and psychological resilience exert a chain-mediating effect on psychological fatigue through athletes’ gratitude (Ye et al., 2016). Perceived social support can positively predict psychological resilience, an important positive psychological trait (Zhu et al., 2024); psychological resilience can reduce negative emotions in individuals and enhance mental health and subjective wellbeing (Jiang et al., 2024; Usborne and Taylor, 2010). Perceived social support can be conceptualized as a critical external resource that facilitates individuals’ adaptive processes. Wills, social support mitigates the negative effects of stress by providing emotional, informational, and instrumental resources, thereby enhancing individuals’ coping capacity (Cohen and Wills, 1985). External resources, such as social support, can be internalized and transformed into psychological resources. From this perspective, social support contributes to the accumulation of internal strengths, including psychological resilience. Furthermore, resilience development frameworks emphasize that resilience is not merely an inherent trait but is shaped through continuous interactions with supportive environments. Therefore, individuals who perceive higher levels of social support are more likely to develop stronger adaptive capacities and resilience. Perceived social support is positioned as an antecedent of psychological resilience in the present study. This implies that individuals who perceive a higher level of social support are more likely to develop greater adaptability and psychological resilience. Therefore, this study positions perceived social support as a precursor to psychological resilience in its theoretical framework. Therefore, the research hypothesis H4: Perceived social support and psychological resilience play a chain mediating role in physical exercise and subjective wellbeing is proposed.

**Figure 1:**
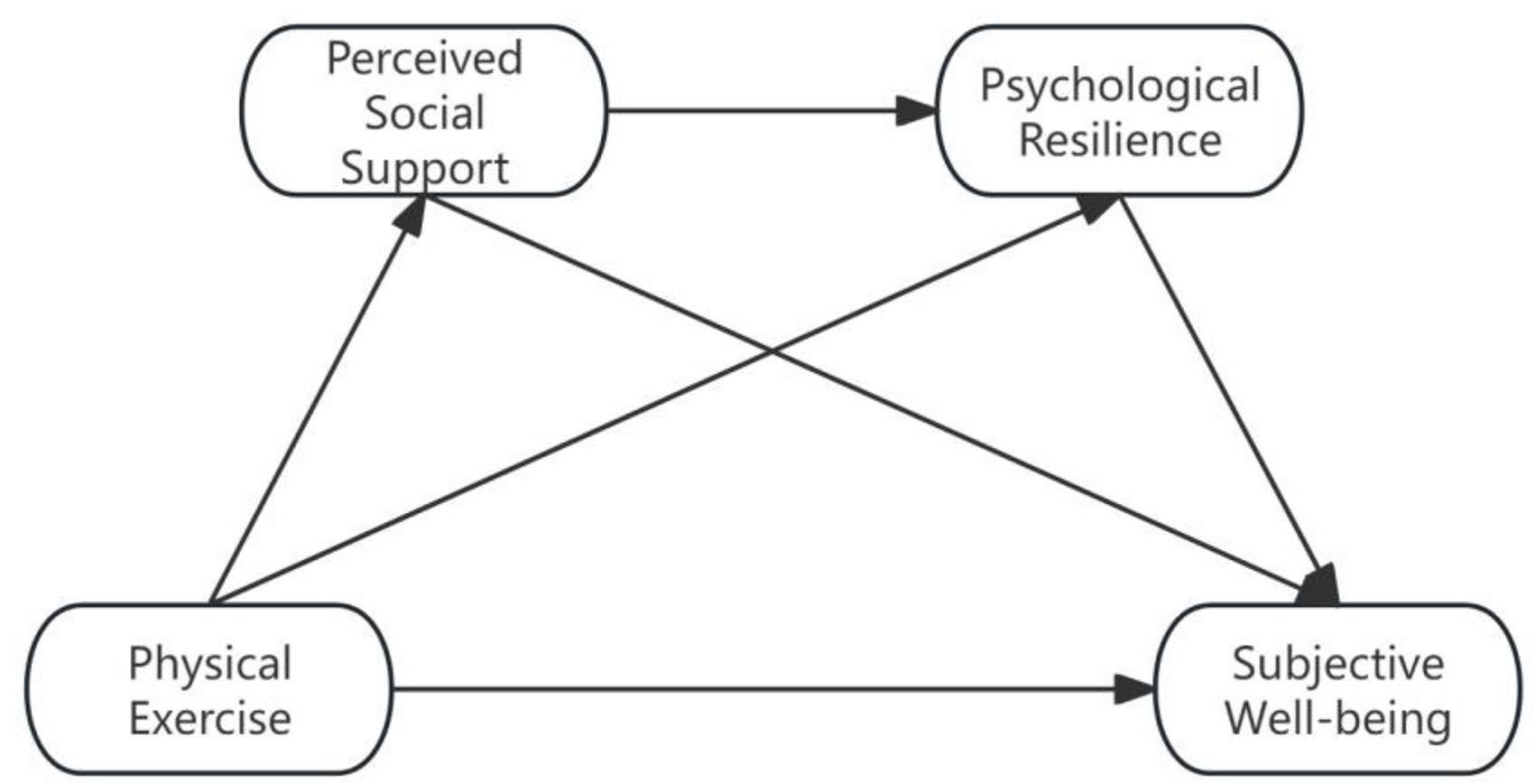
Hypothesized model of physical exercise on subjective wellbeing of college students.

As a result, the research hypothesis model was constructed as shown in Figure 1.

## 3 Research objects and methods

### 3.1 Research object

The focus of this study is the impact of physical exercise on university students’ subjective wellbeing, as well as the chained mediating effects of perceived social support and psychological resilience. To enhance the representativeness of the sample and ensure coverage of university students across different academic years, this study employed a stratified random sampling method based on year of study, selecting students from four different years as the research subjects, and distributed a total of 513 questionnaires. Data were collected through an online questionnaire distributed via university networks and social media platforms in Dalian and Qingdao. The questionnaire was distributed on January 20, 2026, and was collected on February 10, 2026. All participants were adults who obtained verbal informed consent prior to participation and were ensured that they fully understood the study objectives, procedures and potential risks. Of these, 9 invalid questionnaires were excluded, including those with extreme responses, incomplete responses, and responses that did not meet the time constraints. Extreme responses refer to those that were entirely consistent or excessively biased. A total of 504 valid questionnaires were recovered, yielding a response rate of 98.2%. The results of valid questionnaires returned were 504, 243 (48.2%) male and 261 (51.8%) female; in terms of grade, 113 (22.4%) were freshmen, 150 (29.8%) were sophomores, 83 (16.5%) were juniors, and 158 (31.3%) were seniors. This diversity provides a reasonable basis for examining the relationships among the study variables within the sampled population. The specific details of the sample selection are shown in Table 1, and the sample size meets the required standards.

**Table 1:** Sample demographics.

Variable	Categories	Quantities	Percentage
Gender	Male student	243	48.2%
	Female student	261	51.8%
Grade	First-year university student	113	22.4%
	Second-year university student	150	29.8%
	Third-year university student	83	16.5%
	Fourth-year university student	158	31.3%

### 3.2 Research tools

#### 3.2.1 Physical Activity Rating Scale

The Physical Activity Rating Scale complied by Hashimoto (2005) and revised by Deqing (1994), was used to assess the participants’ levels of physical exercise. The scale primarily evaluates exercise volume based on three aspects: intensity, frequency and duration of physical activity. All indicators were scored using a 5-point Likert scale. The total physical activity score is calculated as: Intensity × (Duration − 1) × Frequency, with higher scores indicating higher levels of physical activity. A higher score indicates a higher level of participation in physical activity among students. This scale has been widely used in psychological research and has demonstrated good reliability and validity across different cultural contexts, including studies involving college students in China. The internal consistency Cronbach’s alpha coefficient of the scale in this study was 0.712, with good reliability and validity.

#### 3.2.2 Subjective Wellbeing Scale

The Subjective Wellbeing Scale revised by Jianhua (1996), was used to assess the participants’ levels of wellbeing. The scale comprises 18 items and covers six dimensions: concerns about health, energy, life satisfaction, positive mood, behavioral control, and relaxation versus tension. Higher scores indicate greater wellbeing. The internal consistency Cronbach’s alpha coefficient of the scale in this study was 0.901, with good reliability and validity.

#### 3.2.3 Perceived Social Support Scale

The Perceived Social Support Scale developed by Zimet et al. (1988) was employed. The scale comprises 12 items, divided into three factors: family support, friend support and other support. It uses a 7-point Likert scale; a higher score indicates that the perceived social support has a greater impact on the individual. This instrument has been extensively validated in diverse cultural settings, including Chinese samples, with strong psychometric properties. The internal consistency Cronbach’s alpha coefficient of the scale in this study was 0.807, with good reliability and validity.

#### 3.2.4 Psychological Resilience Scale

The Psychological Resilience Scale developed by Hu and Gan (2008) was used to asses the subjective wellbeing of college students. Consisting of 27 items, it is divided into 5 dimensions: goal orientation, emotional regulation, positive cognition, family support and interpersonal support. Twelve of these items are reverse-scored. The scale uses a 5-point Likert scale, with numbers 1–5 representing levels ranging from low to high. The Cronbach’sαcoefficient is 0.953.

### 3.3 Statistical methods

Data analysis in this study was conducted using SPSS 29.0 statistical software. Firstly, Cronbach’s alpha was used to assess reliability, and Harman’s one-way test was employed to examine common method bias following data collection. Secondly, after importing the data into SPSS, descriptive statistics were used to conduct demographic analyses. Pearson’s correlation coefficient was used to analyze the relationships between physical exercise, subjective wellbeing, perceived social support and psychological resilience. Multivariate regression analysis was conducted using Model 6 in the PROCESS macro, and the significance of the mediating effects was assessed using the Bootstrap test to analyze the study.

## 4 Results

### 4.1 Control and testing of common method bias

As all data in this study were derived from subjective questionnaires, it was necessary to conduct a test for common-method bias. To reduce the potential influence of common method bias, both procedural and statistical remedies were applied in this study. Procedurally, several measures were implemented during data collection, including ensuring anonymity, emphasizing that there were no right or wrong answers, and using standardized instructions to minimize evaluation apprehension. In addition, all measurement instruments used in this study were well-established scales with demonstrated reliability and validity. Statistically, Harman’s single-factor test was conducted as a preliminary diagnostic approach. The results indicated that the first unrotated factor accounted for 37.023% of the total variance, which is below the commonly accepted threshold of 40%, suggesting that common method bias is unlikely to pose a serious threat to the validity of the findings.

### 4.2 Independent sample *t*-test

Independent samples t-test was used to analyze the differences in physical exercise, subjective wellbeing, perceived social support and psychological resilience among college students of different genders, as shown in Table 1. The results indicate that no statistically significant gender differences were found in key variables such as physical exercise, subjective wellbeing, perceived social support and psychological resilience (*p* > 0.05) Based on the descriptive statistics, the differences in mean scores between males and females across all variables were small, and the standard deviations were close to the standard errors, indicating that the sample distribution exhibited a high degree of consistency and stability. To some extent, this result reflects the trend towards a narrowing of gender differences within the contemporary sample population, suggesting that both males and females demonstrate relatively similar levels of development, whether in terms of health behaviors or psychological perceptions. Furthermore, variables such as subjective wellbeing and perceived social support are likely to be influenced more by a combination of individual social contexts, psychological resources and behavioral patterns, rather than being determined by a single demographic variable. Therefore, future research could, whilst controlling for gender, focus on exploring the mechanisms underlying the relationships between physical activity participation, social support and psychological adjustment, in order to identify more explanatory pathways of influence (see Table 2).

**Table 2:** Differences in physical exercise, subjective wellbeing, perceived social support and psychological resilience by gender.

Relevant variable	Gender	N	Mean	Std. deviation	Std. error mean	*p*-value
Physical exercise	Male	243	2.82	0.86	0.0560	0.75
	Female	261	2.88	0.85	0.0524	
Subjective wellbeing	Male	243	3.57	0.84	0.0539	0.95
	Female	261	3.56	0.85	0.0528	
Perceived Social Support	Male	243	4.05	0.90	0.0579	0.36
	Female	261	4.08	0.97	0.0598	
Psychological resilience	Male	243	3.24	0.84	0.0540	0.33

### 4.3 Correlation analysis between variables

Using Pearson’s correlation coefficient, an analysis of the correlations between the main variables was conducted using SPSS 29.0. The results are shown in Table 3. The findings indicate that university students’ subjective wellbeing is significantly positively correlated with physical exercise (*r* = 0.759, *p* < 0.01); perceived social support is significantly positively correlated with psychological resilience (*r* = 0.850, *p* < 0.01); physical exercise was significantly positively correlated with perceived social support (*r* = 0.710, *p* < 0.01) and psychological resilience (*r* = 0.753, *p* < 0.01); Subjective wellbeing was positively correlated with perceived social support (*r* = 0.864, *p* < 0.01) and psychological resilience (*r* = 0.890, *p* < 0.01).

**Table 3:** Pearson correlation coefficient.

Relevant variable	Physical exercise	Subjective wellbeing	Perceived social support	Psychological resilience
Physical exercise	1			
Subjective wellbeing	0.759^**^	1		
Perceived social support	0.710^**^	0.864^**^	1	
Psychological resilience	0.753^**^	0.890^**^	0.850^**^	1

**p < 0.01.

### 4.4 Regression analysis and mediating effects test

The chain mediating effects of perceived social support and psychological resilience between physical exercise and subjective wellbeing were tested and the results are shown in Table 4. The specific steps are as follows: First, a regression model was established with perceived social support as the dependent variable and gender and year group as control variables. The results indicate that, after controlling for covariates, physical exercise significantly and positively predicts perceived social support (*β* = 0.707, *p* < 0.001), suggesting that physical exercise has a positive association with on students’ perceived social support. In the second step, psychological resilience was set as the dependent variable, with gender and year group included as control variables, whilst physical exercise and perceived social support were also included. The results indicate that physical exercise continues to have a significant positive predictive effect on psychological resilience (*β* = 0.304, *p* < 0.001), and that perceived social support significantly and positively predicts psychological resilience (*β* = 0.637, *p* < 0.001). This suggests that physical exercise is associated with the psychological resilience both directly and indirectly via perceived social support. In the third step, subjective wellbeing was taken as the dependent variable, with gender and year group included as control variables, whilst physical exercise, perceived social support and psychological resilience were also included. The results showed that, after controlling for covariates, the direct predictive effect of physical exercise on subjective wellbeing remained significant (β = 0.148, *p* < 0.001); perceived social support significantly and positively predicted subjective wellbeing (*β* = 0.349, *p* < 0.001); psychological resilience also significantly and positively predicted subjective wellbeing (β = 0.478, *p* < 0.001). Furthermore, after controlling for gender and year group, the total effect of physical exercise on subjective wellbeing was significant (*β* = 0.756, *p* < 0.001).

**Table 4:** Regression analysis of variable relationships.

Equation of regression		Overall fit index			Significance of regression coefficient		
Result variable	Variable of prediction	*R*	*R*^2^	*F*	*β*	*t*	*p*
Perceived social support	Physical exercise	0.711	0.506	170.577^***^	0.707	22.377^***^	0.000
	Gender				−0.009	−0.285	0.776
	Grade				0.041	1.306	0.192
Psychological resilience	Physical exercise	0.878	0.770	417.749^***^	0.304	9.959^***^	0.000
	Perceived social support				0.637	20.869^***^	0.000
	Gender				−0.037	−1.710	0.088
	Grade				−0.018	−0.811	0.418
Subjective wellbeing	Physical exercise	0.919	0.844	539.012^***^	0.148	5.389^***^	0.000
	Perceived social support				0.349	10.116^***^	0.000
	Psychological resilience				0.478	12.965^***^	0.000
	Gender				−0.013	−0.744	0.457
	Grade				0.037	2.072	0.039
Subjective wellbeing	Physical exercise	0.762	0.580	230.518^***^	0.756	25.963^***^	0.000
	Gender				−0.037	−1.264	0.207
	Grade				0.055	1.907	0.057

***p < 0.001, *p < 0.05.

**Figure 2:**
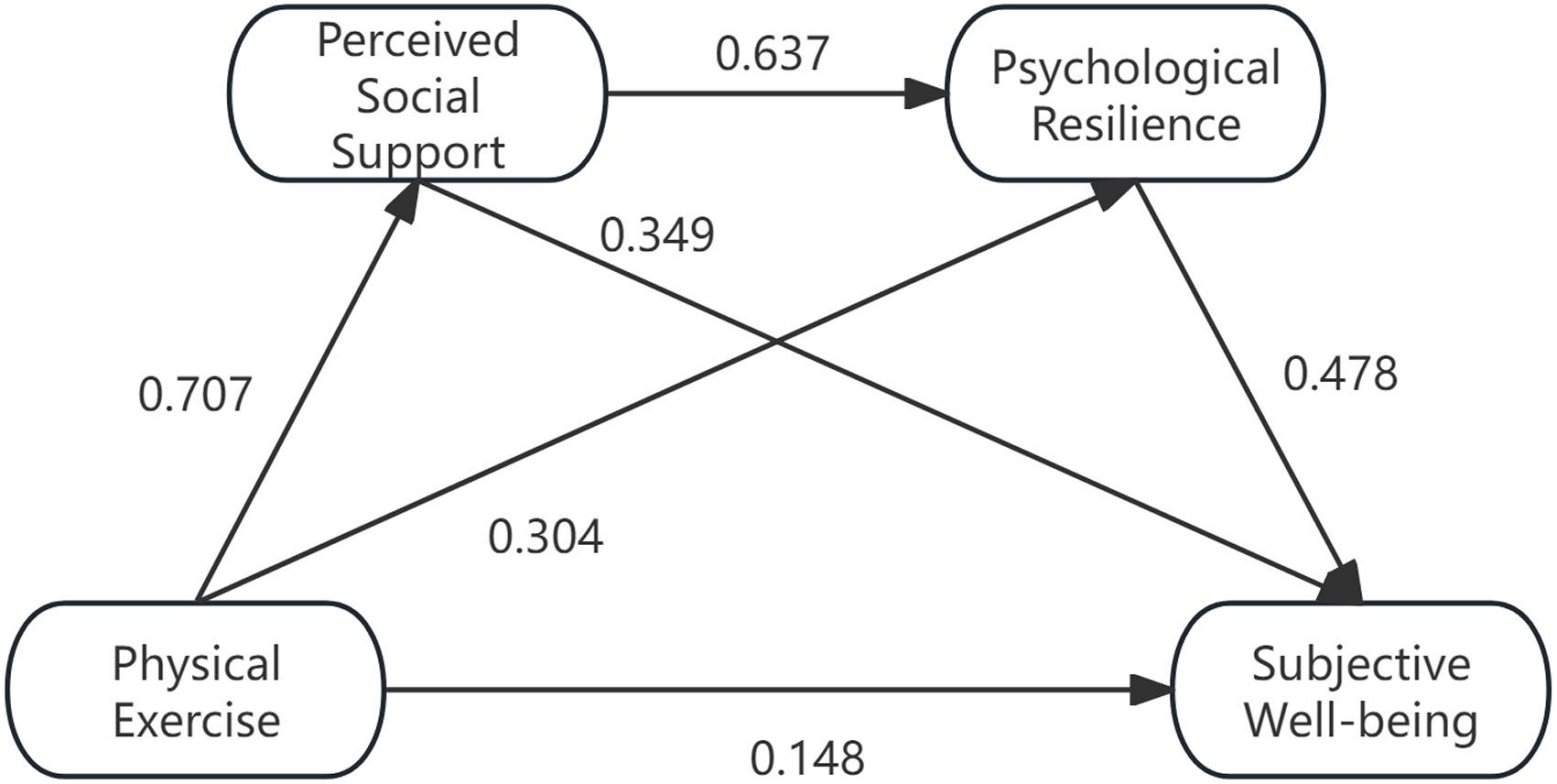
A mediating model of physical exercise affecting students’ subjective wellbeing. **p < 0.01; ***p < 0.001.

Bootstrap test was used to repeat the sampling 5,000 times to test the mediating effects of perceived social support and psychological resilience between physical exercise and subjective wellbeing and the confidence intervals, respectively, as shown in Table 5. The results showed that physical exercise produced a total effect value of 0.7514 on subjective wellbeing, and the 95% confidence intervals for the mediating effects of perceived social support and psychological resilience did not contain 0 (LLCL = 0.6949, ULCL = 0.8079), which indicated that the total effect of physical exercise on subjective wellbeing as well as the mediating effects of the two variables were significant. The value of the direct effect of physical exercise on subjective wellbeing is 0.1476 (direct path), Bootstrap 95% confidence interval does not contain 0 (LLCL = 0.0940, ULCL = 0.2012), which indicates that there is a significant direct effect of physical exercise on subjective wellbeing, with an effect value of 19.6% of the total effect. Based on the above results, the following model diagram is derived as shown in Figure 2. In addition to statistical significance, the magnitude of the indirect effects was also considered. The standardized indirect effects obtained via the Bootstrap procedure were generally in the small to moderate range according to commonly accepted benchmarks. Although not large, these effects are not negligible, suggesting that the identified mediation pathways may have practical relevance.

**Table 5:** Proportion of the mediating effect.

Effect	Trails	Effect value	Standard error	LLCL	ULCL	Effect ratio
Total effect		0.7514	0.0288	0.6949	0.8079	100%
Direct effect	Direct path	0.1476	0.0273	0.0940	0.2012	19.6%
Total indirect effect		0.6038	0.0261	0.5534	0.6560	80.4%
Indirect effect	Ind1 (physical exercise → perceived social support → Subjective wellbeing)	0.2476	0.0260	0.1977	0.3000	33.0%
	Ind2 (physical exercise → psychology resilience → subjective wellbeing)	0.1426	0.0169	0.1097	0.1762	19.0%
	Ind3 (physical exercise → perceived social support → psychology resilience → subjective wellbeing)	0.2137	0.0206	0.1756	0.2563	28.4%

## 5 Discussion

### 5.1 The relationship between physical exercise and subjective wellbeing

The results of the study found a significant positive correlation between physical exercise and subjective wellbeing among university students, which verified hypothesis H1. The findings revealed a furthermore, even after controlling for mediating variables, the positive predictive effect of physical exercise on subjective wellbeing remained significant. This finding is broadly consistent with the conclusions of previous research and further underscores the significant value of physical exercise in promoting mental health and positive psychological experiences. From a theoretical perspective, physical exercise is not merely a form of physical activity, but also an important means of psychological regulation. The field of cognitive neuroscience has gradually unveiled the physiological mechanisms through which physical exercise influences subjective wellbeing, and the ways in which exercise alters brain structure and optimizes brain function are attracting increasing attention from researchers. The shared or overlapping network system comprising the prefrontal cortex, hippocampus, amygdala, ventral striatum, and nucleus accumbens, together with the neurotransmitters and hormones such as endorphins, dopamine, and serotonin produced by these regions, forms an integrated circuit capable of sensing emotional responses (Qiao and Fan, 2020). Existing research indicates that physical exercise exerts varying degrees of influence on the aforementioned brain regions, and it is increasingly recognized that physical exercise promotes the production of brain-derived neurotrophic factor (BDNF), often referred to as ‘brain cell fertilizer’.

According to theories in positive psychology, subjective wellbeing is a comprehensive reflection of an individual’s overall cognitive evaluation of their quality of life and their emotional experiences, encompassing life satisfaction as well as the frequency and intensity of positive emotions. Physical exercise provides a crucial foundation for enhancing university students’ subjective wellbeing through multiple mechanisms, including the improvement of physiological functions, the stimulation of positive emotional experiences, and the promotion of social interaction (Buecker et al., 2021). On the one hand, physical exercise can promote the secretion of neurotransmitters (such as endorphins and dopamine) through physiological mechanisms, thereby alleviating anxiety, stress and negative emotions, enhancing an individual’s sense of pleasure and emotional stability, and making it easier for them to generate positive emotions and achieve a higher level of wellbeing (Dai and Yao, 2012). On the other hand, physical exercise possesses significant psychological regulatory functions. As college students are at a critical stage of their personal development, facing multiple stressors such as academic pressure, employment anxiety and social adaptation, physical exercise provides an effective avenue for emotional release and stress relief. Through the experiences of focus and achievement during exercise, individuals can temporarily escape stressful situations, enhance their sense of self-efficacy and control over their lives, thereby improving life satisfaction and overall wellbeing.

Furthermore, from a sociocultural perspective, physical exercise is often characterized by a distinct sense of interaction and community (Belon et al., 2016). Whether in team sports or group physical activities, these provide university students with greater opportunities for social interaction and emotional exchange. Such social interaction not only enhances individuals’ sense of belonging and social connectedness, but also improves their emotional wellbeing and sense of life’s meaning through positive interpersonal interactions. Particularly within the campus environment, physical activities often serve as a vital medium for students to forge friendships and develop a sense of group identity; this sense of social connectedness further reinforces their experience of happiness. From a developmental perspective, university students are at a stage of rapid psychological and social role development, and their subjective wellbeing is easily influenced by factors such as academic competition, career planning and social adaptation. As a low-cost and sustainable health behavior, physical exercise not only promotes physical health but also helps individuals establish positive lifestyles and psychological resources, thereby forming a more stable foundation for wellbeing in the long term (Huijun and Qingting, 2012).

### 5.2 Mediating role of perceived social support in physical exercise and subjective wellbeing

It was found that the effect of physical exercise on subjective wellbeing can also be realized through an indirect pathway, perceived social support, validating hypothesis H2. The finding that perceived social support predicts subjective wellbeing is consistent with previous research. Perceived social support is a predictor of subjective wellbeing. This finding further elucidates the socio-psychological mechanisms through which physical exercise is related to an individual’s experience of wellbeing. It refers to an individual’s subjective perception and evaluation of support resources from family (Mao and Zhao, 2023), friends and significant others; it not only reflects the individual’s actual social network but also embodies their emotional experiences and sources of security within social interactions. This study found that physical exercise can enhance university students’ subjective wellbeing by increasing their perceived social support. This suggests that physical exercise is related to the experience of wellbeing not only through physiological and emotional mechanisms, but also by strengthening social connections and the perception of social support.

Firstly, physical exercise provides university students with important opportunities for social interaction, helping to enhance their perception of social support. Through participation in sporting activities, individuals are not only able to forge new social relationships, but also receive emotional, informational and evaluative support through these interactions, thereby strengthening their positive perception of social relationships. When individuals experience encouragement, cooperation and recognition from peers in sporting contexts, they are more likely to develop a sense of being ‘supported’ and ‘accepted’; this positive experience of social relationships significantly enhances their overall perception of social support. Consequently, physical exercise, to a certain extent, establishes a crucial contextual foundation for university students to obtain social support, making it easier for them to form stable social networks within the campus environment.

Secondly, social support is a vital psychological resource that influences subjective wellbeing. According to the stress-buffer model, social support acts as a protective factor when individuals face stress or challenges; by providing emotional comfort, practical guidance and tangible assistance, it mitigates the adverse effects of negative events on mental health. For university students at a critical stage of their personal development, who face multiple challenges in terms of academic pressure, interpersonal relationships and future prospects, a higher level of perceived social support can enhance their sense of security and belonging, making it easier for them to maintain a positive emotional state when facing stressful situations. When university students experience greater social support, they tend to describe themselves in more positive terms, developing a higher sense of self-esteem; consequently, this positive self-evaluation enhances their subjective wellbeing (Feng et al., 2012). Therefore, perceived social support can promote the development of subjective wellbeing through two mechanisms: emotional regulation and cognitive evaluation.

What’s more, physical exercise enhances subjective wellbeing by increasing the perception of social support, thereby illustrating the vital role of healthy behaviors in the development of psychosocial resources. Physical exercise is not merely a form of physical activity, but rather a lifestyle with social dimensions; it encourages individuals to actively engage in social interactions and, through these interactions, gain emotional validation and social connection (Shabbir et al., 2025). This sense of social connection not only helps to strengthen an individual’s sense of social belonging but also fosters a more positive evaluation of the social environment, thereby further enhancing their experience of wellbeing. Consequently, by promoting social interaction and the perception of social support, physical exercise establishes a vital foundation of psychological resources for university students, enabling them to maintain a more positive emotional state and a higher level of wellbeing in both their studies and daily lives.

### 5.3 The mediating role of psychological resilience in physical exercise and subjective wellbeing

It was also found that the effect of physical exercise on subjective wellbeing can also occur indirectly on subjective wellbeing by promoting psychological resilience, testing hypothesis H3. The predictive effect of psychological resilience on subjective wellbeing is consistent with the findings of previous studies.

During physical exercise, university students encounter pressures associated with learning motor skills, challenges posed by competitors, and the resulting feelings of frustration. Whilst these may have a negative impact on students’ mental health in the short term, successfully overcoming these issues through their own efforts or with the support of peers and teachers enables them to develop a more resilient mindset and enhance their problem-solving abilities. Consequently, they are better equipped to effectively cope with and adapt to stressful situations in daily life, maintaining a positive outlook and emotional state. Whether in adversity or under less stressful conditions, psychological resilience enables individuals to remain optimistic, positive and energetic in their daily lives, thereby maintaining and enhancing their sense of wellbeing (Wang and Wang, 2013).

Firstly, physical exercise plays a significant role in fostering psychological resilience. From a developmental perspective, psychological resilience is not an innate, fixed trait, but rather a psychological capacity that is gradually formed and developed through an individual’s interaction with their environment. As a challenging and goal-oriented activity, physical exercise provides individuals with situations in which they can continually face challenges, adjust their strategies and achieve personal breakthroughs. For example, during sports training or competition, individuals often need to overcome a variety of challenges, such as physical fatigue, technical difficulties and competitive pressure. This repeated cycle of facing challenges and achieving success can enhance an individual’s perseverance, resilience and self-regulation skills. When individuals overcome difficulties through their own efforts and experience progress or success, their sense of self-efficacy and control is further strengthened; these positive psychological experiences help to reinforce their psychological resilience.

Secondly, psychological resilience enables individuals to maintain a positive cognitive evaluation and emotional state when facing stress and adversity (Xing and Ge, 2025), thereby reducing the impact of negative emotions on their sense of wellbeing. College students face multiple pressures in areas such as academic competition, interpersonal relationships and future career development. Without adequate psychological resilience, individuals are prone to experiencing negative emotions such as anxiety and helplessness in stressful situations, which in turn reduces their life satisfaction and overall sense of wellbeing. Conversely, individuals with high psychological resilience typically view challenges in a more positive light and achieve psychological adaptation through effective emotional regulation and problem-solving strategies, thereby enhancing their subjective wellbeing. Consequently, psychological resilience plays a significant role in promoting individual mental health and the development of a sense of wellbeing.

What’s more, physical exercise promotes subjective wellbeing by enhancing psychological resilience, thereby demonstrating the significant value of healthy behaviors in the accumulation of an individual’s psychological resources. Physical exercise not only improves physical health but also enhances an individual’s psychological adaptability through the continuous pursuit of goals, the strengthening of willpower, and the process of emotional regulation, enabling individuals to face the pressures of study and daily life with greater composure and confidence. The accumulation of these psychological resources helps individuals develop a more positive attitude towards life and a more stable emotional state, thereby enhancing their overall life satisfaction and sense of wellbeing. Consequently, physical exercise is not only an important lifestyle choice for promoting physical health, but also a vital means of cultivating psychological resilience and enhancing psychological wellbeing.

### 5.4 Chain mediation between perceived social support and subjective wellbeing

It was also found that perceived social support and psychological resilience, in addition to their respective individual mediating roles between physical exercise and subjective wellbeing, both could also chain mediate between physical exercise and subjective wellbeing, validating hypothesis H4. The impact of physical exercise on university students’ subjective wellbeing is not the result of a single mechanism, but rather takes effect gradually through the interaction between social resources and individual psychological resources, revealing the multi-level mechanisms by which physical exercise promotes psychological wellbeing. The social support university students receive through physical exercise is a key factor influencing psychological resilience. A relatively stable and systematic association exists between students’ social support and psychological resilience (Song et al., 2018), confirming that the perception of social support can enhance an individual’s level of psychological resilience (Herrman et al., 2011).

Firstly, physical exercise can provide a vital external resource base for the development of psychological resilience by enhancing university students’ perception of social support. According to social support theory, social support not only directly improves an individual’s emotional experience but also provides emotional care, informational assistance and practical support, thereby enhancing the individual’s sense of psychological security and belonging when facing difficulties. When individuals perceive understanding, encouragement and support from others within their social relationships, they are more likely to develop a positive self-image and a stable emotional state; such a positive social context helps to foster the formation and development of psychological resilience. For university students, physical exercise typically exhibits strong interactive and group-oriented characteristics. When individuals consistently experience support and acceptance from their social environment, their confidence and adaptive capacity in the face of pressure and challenges are consequently strengthened, providing a vital foundation of social resources for the enhancement of psychological resilience.

Secondly, recognizing the role of social support in fostering psychological resilience further contributes to an increase in subjective wellbeing. From the perspective of psychological resource integration, psychological resilience relies to a certain extent on the support provided by external support systems. When individuals receive stable emotional and resource support within their social networks, they are better able to maintain a positive psychological state when facing stress or setbacks, and adopt more effective coping strategies. Specifically, a higher level of perceived social support can enhance an individual’s sense of security and self-worth, enabling them to face difficult situations with greater confidence and composure; this positive psychological state helps individuals develop stronger psychological resilience. Once psychological resilience is enhanced, individuals are able to adapt effectively to situations such as academic pressure, interpersonal difficulties or uncertainty regarding future development through positive cognition and emotional regulation. This reduces the occurrence of negative emotions, enhances life satisfaction and positive emotional experiences, and ultimately improves overall subjective wellbeing (White and Bennie, 2015).

What’s more, the chain-mediated pathway of ‘social support–psychological resilience’ reflects the process by which external social resources are transformed into internal psychological resources. Physical exercise creates rich social interaction contexts for university students, making it easier for them to obtain support and recognition from peers, friends and significant others. This social support not only directly influences individuals’ emotional experiences but also enhances their psychological resilience at a deeper level. As external social support is gradually internalized as an individual’s psychological resource, the individual is able to maintain a positive and stable psychological state when facing various pressures and challenges, thereby fostering a higher level of subjective wellbeing. Consequently, the chain-mediation effect identified in this study reveals the dynamic process through which physical exercise is positively associated with university students’ experience of wellbeing: physical exercise first enhances an individual’s perception of social support, thereby promoting the development of psychological resilience, and ultimately leading to an improvement in subjective wellbeing.

### 5.5 Practical implications and intervention strategies

As shown in the table, three distinct indirect pathways linking physical exercise to subjective wellbeing were observed, with specific effect sizes and underlying mechanisms detailed below: The Ind1 pathway (physical exercise → perceived social support → subjective wellbeing) accounted for 33.0% of the total effect. This indicates that physical exercise strengthens individuals’ perceived social support, which in turn directly contributes to improvements in subjective wellbeing. The large proportion of this effect suggests that social support derived from exercise participation is a key driver of enhanced wellbeing. The Ind 2 pathway (physical exercise → psychological resilience → subjective wellbeing) constituted 19.0% of the total effect. This shows that physical exercise directly enhances psychological resilience, which then promotes greater subjective wellbeing. The notable contribution of this pathway highlights the role of exercise in fostering personal resilience as a means of improving wellbeing. Finally, the Ind3 serial mediation pathway (physical exercise → perceived social support → psychological resilience → subjective wellbeing) explained 28.4% of the total effect. In this sequential pathway, physical exercise first boosts perceived social support, which then facilitates the development of psychological resilience, ultimately leading to higher subjective wellbeing. This reflects a stepwise process in which exercise-related social gains build the psychological resources needed to support wellbeing. Overall, The findings of this study provide actionable implications for university administrators, coaches, and student support practitioners by translating the identified chain mechanism into concrete intervention strategies. First, exercise program design should emphasize structured social interaction rather than individual participation alone. Universities are encouraged to implement group-based formats (e.g., team sports, fitness classes, peer-led sessions) to strengthen perceived social support during physical activity. Second, institutions can embed explicit social support mechanisms within exercise contexts. For example, pairing systems, peer mentoring, or small-group training formats can be systematically incorporated to enhance interpersonal connection and support perception. Third, practitioners should intentionally integrate resilience-building components into physical activity programs. This can include goal-setting, progressive challenges, and constructive feedback to facilitate adaptive coping and psychological growth through exercise. Fourth, at the institutional level, universities should adopt an integrated intervention framework that simultaneously targets physical activity, social connectedness, and psychological adaptation. This may involve coordinated efforts across departments (e.g., sports services, counseling centers, and academic units) to create a supportive and sustainable wellbeing ecosystem. Overall, these findings suggest that effective wellbeing interventions should move beyond increasing exercise participation alone and instead leverage the synergistic effects of social support and psychological resilience embedded within physical activity contexts.

## 6 Conclusion

Through data analysis, the following conclusions can be drawn: (1) Physical exercise can positively predict college students’ subjective wellbeing; (2) Physical exercise can mediate college students’ subjective wellbeing through perceived social support and psychological resilience, respectively; (3) Physical exercise can affect college students’ subjective wellbeing through perceived social support and psychological resilience in a chain. Three pathways of mediating effects: physical exercise → perceived social support → subjective wellbeing (path 1), physical exercise → psychological resilience → subjective wellbeing (path 2), and physical exercise → perceived social support → psychological resilience → subjective wellbeing (path 3).

## 7 Limitations and future directions

This research, while providing useful insights, has some limitations that must be recognized. There are several limitations to this study. Firstly, the cross-sectional design limits causal inference. Although the proposed relationships are theoretically grounded, the findings should be interpreted as associations rather than causal effects. Moreover, the potential reciprocal relationship between perceived social support and psychological resilience remains unexplored. Future research should employ longitudinal or experimental designs, particularly cross-lagged panel designs, to establish temporal precedence and examine causal mechanisms, including reciprocal dynamics. Secondly, the sample was drawn from a specific population, which may limit the generalizability of the findings to broader populations. Differences in educational, cultural, or institutional contexts could influence the observed patterns. Future studies should recruit more diverse and representative samples across different educational levels, geographic regions, and cultural contexts. Replication studies in varied settings are needed to assess the robustness and external validity of the observed associations. Thirdly, all variables were measured using self-report instruments, which may introduce common method bias. Although procedural remedies and Harman’s single-factor test were applied, the potential for inflated associations due to shared method variance cannot be entirely ruled out. Future research should incorporate multi-method assessments and advanced statistical techniques to reduce common method variance and further control for method effects. Fourthly, omission of demographic and socioeconomic covariates. Important covariates—such as academic stress, urban versus rural household registration status, socioeconomic status, field of study, and personality traits—were not controlled for in the chained mediation model. Although data on some of these variables were collected, they were not included as statistical controls. This omission may introduce confounding effects, potentially inflating or obscuring the observed relationships. Future research should systematically include these variables as control variables or moderators in analytical models to isolate the unique effects of physical exercise and clarify conditional mechanisms. Exploring how these variables moderate or mediate the chained pathways would also provide a more nuanced understanding of the relationships.

## Data Availability

The original contributions presented in the study are included in the article/supplementary material, further inquiries can be directed to the corresponding author.
